# SARS-CoV-2 infection risk is higher in vaccinated patients with inflammatory autoimmune diseases or liver transplantation treated with mycophenolate due to an impaired antiviral immune response: results of the extended follow up of the RIVALSA prospective cohort

**DOI:** 10.3389/fimmu.2023.1185278

**Published:** 2023-07-20

**Authors:** Manuela Rizzi, Stelvio Tonello, Cristiana Brinno, Erika Zecca, Erica Matino, Micol Cittone, Eleonora Rizzi, Giuseppe Francesco Casciaro, Davide D’Onghia, Donato Colangelo, Rosalba Minisini, Mattia Bellan, Luigi Mario Castello, Annalisa Chiocchetti, Mario Pirisi, Cristina Rigamonti, Daniele Lilleri, Federica Zavaglio, Federica Bergami, Daniele Sola, Pier Paolo Sainaghi

**Affiliations:** ^1^ Department of Health Sciences, Università del Piemonte Orientale, Novara, Italy; ^2^ Department of Translational Medicine, Università del Piemonte Orientale, Novara, Italy; ^3^ CAAD, Center for Autoimmune and Allergic Diseases, and IRCAD (Interdisciplinary Research Center of Autoimmune Diseases), Università del Piemonte Orientale (UPO), Novara, Italy; ^4^ Department of Internal Medicine and COVID-19 Unit, AOU “Maggiore della Carità”, Novara, Italy; ^5^ Division of Emergency Medicine and COVID-19 sub-intensive unit, AOU “Maggiore della Carità”, Novara, Italy; ^6^ Internal Medicine and Rheumatology Unit, AOU “Maggiore della Carità”, Novara, Italy; ^7^ Division of Internal Medicine, Azienda Ospedaliera “SS. Antonio e Biagio e Cesare Arrigo”, Alessandria, Italy; ^8^ Unit of Microbiology and Virology, Fondazione IRCCS Policlinico San Matteo, Pavia, Italy

**Keywords:** SARS-CoV-2, mycophenolate, inflammatory autoimmune diseases, liver transplantation, anti-COVID-19 mRNA vaccination

## Abstract

**Background:**

A relevant proportion of immunocompromised patients did not reach a detectable seroconversion after a full primary vaccination cycle against SARS-CoV-2. The effect of different immunosuppressants and the potential risks for SARS-CoV-2 infection in these subjects is largely unknown.

**Methods:**

Patients from the Rivalsa prospective, observational cohort study with planned anti SARS-CoV-2 third dose mRNA vaccination between October and December 2021 were asked to participate to this follow-up study. Patients were asked about eventual confirmed positivity to SARS-CoV-2 infection within 6 months from the third dose and to undergo a blood draw to evaluate seroconversion status after the additional vaccine shot.

**Results:**

19 out of 114 patients taking part in the survey developed a confirmed SARS-CoV-2 infection; we identified mycophenolate treatment as an independent predictor of an increased risk of infection even after the third vaccine dose (OR: 5.20, 95% CI: 1.70-20.00, p=0.0053). This result is in agreement with the *in vitro* evidence that MMF impairs both B and T lymphocytes driven immune responses (reduction both in memory B cells producing anti-spike antibodies and in proliferating CD4+ and CD8+ T cells).

**Conclusions:**

Immunocompromised patients need an additional vaccine administration to reach a detectable seroconversion, thus fostering a more personalized approach to their clinical management. Moreover, patients undergoing mycophenolate treatment show a specific increased infection risk, with respect to other immunosuppressants thus supporting a closer monitoring of their health status.

## 1 Introduction

In late 2019, a novel member of the Coronavirus family named SARS-CoV-2 (severe acute respiratory syndrome coronavirus 2) was first detected in China and rapidly spread all around the world. Infected individuals develop COVID-19, a very heterogeneous disease, which clinical manifestations range from asymptomatic disease to life-threatening conditions, mainly characterized by severe pneumonia and multiorgan failure (1–3). COVID-19 quickly became a global healthcare concern, with the high mortality rate observed in frail populations (i.e. elderly, patients with chronic diseases, cancer patients), thus fostering the research for effective drug treatments and vaccines (4–6).

The lack of specific and approved drug treatments assuring clinical recovery from the disease still fosters the development of even more efficient vaccine platforms, as mass vaccination campaign still represents the only available resource to limit COVID-19 diffusion (7–9). In western countries, the approved vaccines most commonly used to sustain the mass vaccination campaign were the mRNA-based ones (BNT162b2 (Pfizer-BioNTech) and mRNA-1273 (Moderna)). During the first phase of the immunization campaign, both vaccines were administered as a series of two doses (21 (BNT162b2) or 28 (mRNA-1273) days apart) and many studies on healthy individuals showed the induction of a strong B and T cell response, without significant differences among the two vaccines (9–11).

There is evidence that vaccine efficacy/effectiveness against infection decreases over time in healthy subjects, falling down below 50% starting from 5 months after vaccination (12, 13), with a decline rate more evident in the elderly, thus fostering the need of a third dose administration to maintain high protection levels in the general population. In this context frail patients, such as immunocompromised ones, have been shown to experience a strong decrease in their IgG and neutralizing antibody titers, thus remaining at higher risk of adverse outcomes (6, 12, 14, 15). Due to the poor seroconversion observed in autoimmune and cancer patients, as well as in solid organ transplant recipients (6, 14, 16–18), they have been prioritized, along with the elderly (6, 12, 14, 16), in the booster vaccination campaign (third and even fourth vaccine administration) in Italy as well as in many other countries all around the world (19, 20).

In a previous prospective observational study (21) our group described, after the completion of the two-doses primary vaccination cycle with the approved mRNA vaccines (BNT162b2 and mRNA-1273), a limited, but relevant, lack of seroconversion in immunosuppressed inflammatory-autoimmune patients and liver transplant recipients. In the original work, we enrolled 131 patients undergoing immunosuppressive treatment, obtaining a complete 90 days follow-up for 119 of them. 100 out these 119 patients showed a detectable IgG response to anti-COVID-19 vaccination and we identified mycophenolate mofetil (MMF) treatment and the compresence of an active neoplasia as the major predictors of such suboptimal response to vaccination. In this prospective, observational study, we further followed up these patients and evaluated their seroconversion rate after the third vaccine dose and the risk of COVID-19 infection.

## 2 Materials and methods

### 2.1 Patients

**Figure 1:**
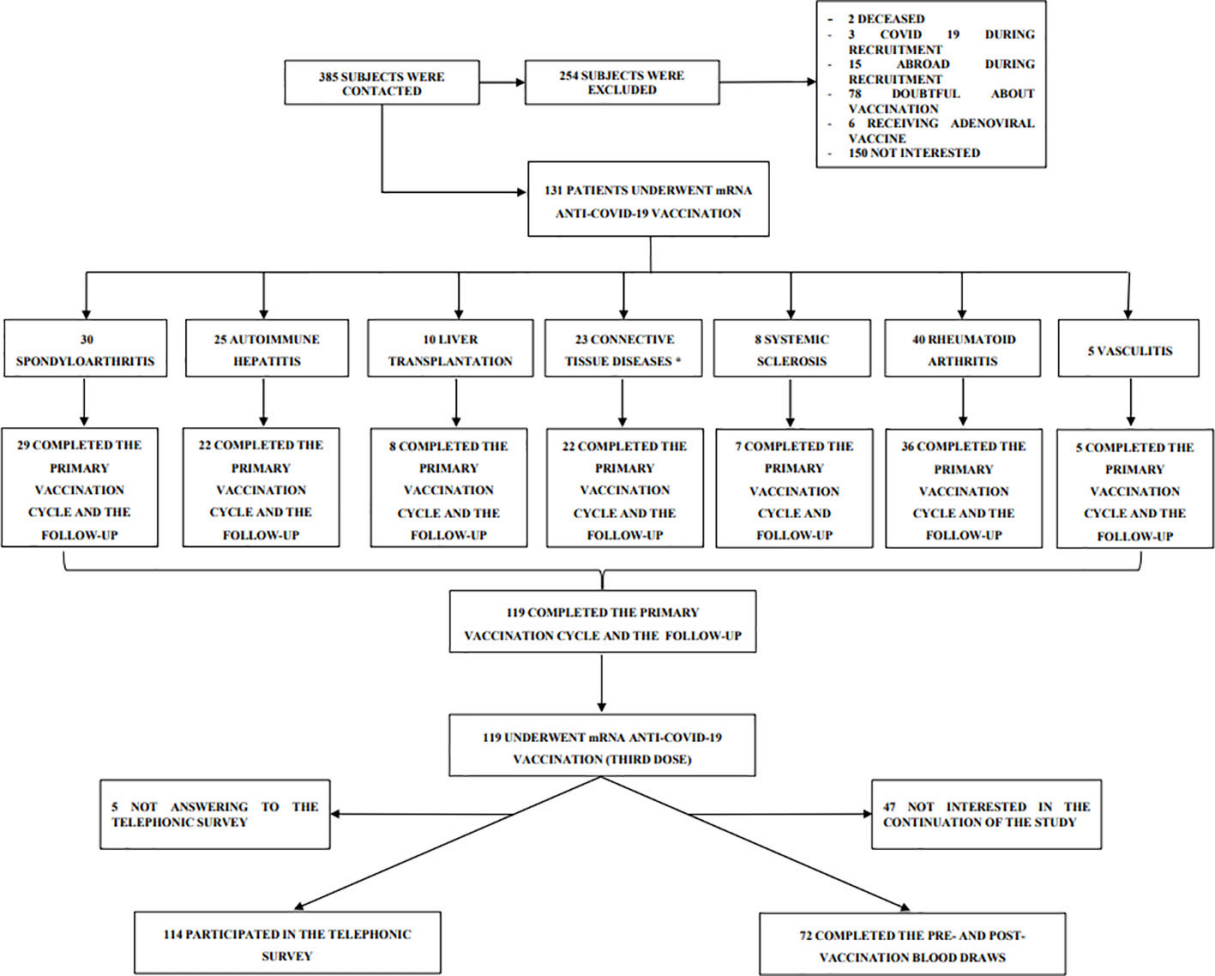
Study design. Flow chart of patient enrollment. *Ten patients have overlapping diseases.

In this prospective, observational cohort study (acronym Rivalsa) we followed up the immunosuppressed autoimmune/liver transplanted patients already described in a previous study focused on seroconversion rate after the conclusion of the primary vaccination cycle (first and second dose) (21). Patients of the Rivalsa cohort with planned third mRNA vaccination (first booster dose) between October 2021 and December 2021 were asked to take part to this follow-up study. Inclusion and exclusion criteria for immunosuppressed patients attending either the Rheumatology or the Hepatology Units of “Maggiore della Carità” University Hospital in Novara (Italy) are reported in **Table 1**. The study was conducted in accordance with the Declaration of Helsinki and the protocol was approved by the local ethical committee (CE 72/21). All patients were asked to undergo a blood draw for anti-SARS-CoV-2 spike protein antibody detection before (t0) as well as 30 (t30) and 90 (t90) days after vaccination. Baseline blood draw (t0) was collected during the week before the planned third dose administration. Moreover, all patients have been contacted by phone 6 months after the vaccination and asked about their COVID-19 infection status in the previous 6 months. All patients received the third vaccination with BNT162b2 (Pfizer-BioNTech) or mRNA-1273 (Moderna) vaccines in clinical practice, according to local protocol and vaccination schedule (**Figure 1**).

**Table 1:** Enrollment criteria.

Inclusion criteria	Exclusion criteria
Age > 18 years	SARS-CoV-2 infection at the time of enrollment
Diagnosis of: - spondyloarthritis, - autoimmune hepatitis, - rheumatoid arthritis, - connective tissue diseases, - vasculitis, - liver transplantation	Concomitant immunodeficiency
Ongoing chronic immunosuppressive therapy	Unwillingness to undergo COVID-19 booster vaccination
Planned mRNA-based (BNT162b2 or mRNA-1273) anti-SARS-CoV-2 booster vaccination between October 2021 and December 2021	Planned adenoviral-based anti-SARS-CoV-2 booster vaccination

### 2.2 Endpoints definition

Predefined endpoints were the following:

1) Assessment of the infection rate among vaccinated immunosuppressed patients;
2) Identification of predictors of infection risk after the first booster vaccination in immunosuppressed patients;
3) Assessment of the IgG anti-SARS-CoV-2 titer in response to mRNA vaccine third dose in immunosuppressed patients.

### 2.3 Blood sample collection

For anti-SARS-CoV-2 antibody quantification, patients underwent blood sampling before the third dose of anti-SARS-CoV-2 vaccination (t0) and after 30 and 90 days from vaccine administration (t30 and t90, respectively). Blood samples were collected by venous puncture using EDTA as anticoagulant and immediately centrifuged. The obtained blood fractions were stored at -80°C until analysis. For ex-vivo experiments, patients and healthy controls underwent blood sampling once after vaccine administration. Blood samples were collected by venous puncture using heparin as anticoagulant and immediately processed to isolate peripheral blood mononuclear cells (PBMCs).

### 2.4 Anti-SARS-CoV-2 antibody quantification

IgG anti-spike protein antibodies were determined using a CE-IVD commercial kit (EUROIMMUN Medizinische Labordiagnostika AG Anti-SARS-CoV-2 QuantiVac ELISA (IgG), Lübeck, Germany) (21–23). This quantitative kit has been chosen as it shows a great correlation with conventional as well as surrogate neutralizing antibodies assays (24–26). To perform antibodies’ quantification, samples were diluted 1:101 according to the provided protocol. Optical densities at 450 nm were recorded on a Victor X4 microplate reader (Perkin Elmer, Whaltman, MA, USA) and antibody titers were quantified by comparison with a calibration curve prepared using human IgG (0-120 RU/ml).

### 2.5 Infection risk evaluation

To calculate the infection risk after the third vaccine dose, all the patients that completed the follow-up of the previous study (21) were contacted and asked to take part in a telephonic survey to collect data about their positivity to SARS-CoV-2 infection (confirmed by RT-PCR or third generation antigenic tests on nasal swabs) following the third vaccine administration.

### 2.6 Spike-specific T- and B-cell responses

PBMCs were isolated from heparin-treated blood. Spike-specific T-cell response was evaluated using ex-vivo ELISpot assay after culture with Spike (S)-specific peptide pool (27) using membrane-bottomed 96-well plates (Multiscreen-IP, Merck Millipore, Germany) coated with anti-interferon (IFN)-γ monoclonal capture antibody from Human IFN-γ ELISpot kits (Diaclone, France) at 4°C overnight. After 2 h blocking with culture medium, 2×10^5^ cells/100 μL/well were stimulated overnight with antigens, while phytohemagglutinin (PHA, 5 μg/ml, Sigma-Aldrich) and medium alone were used as controls. After multiple washes, anti-IFN–γ biotinylated antibody was added and incubated overnight at 4°C. After 60 min incubation with streptavidin–alkaline phosphatase conjugate, 5-bromo-4-chloro-3-indolyl phosphate/nitro blue tetrazolium (BCIP/NBT) substrate was added for 20 min at room temperature. Plates were washed under running water and kept overnight at room temperature before spot counting with AID ELISPOT reader system from Autoimmun Diagnostika GmbH (Strasburg, Germany). Results equal to or higher than 10 IFN-γ SFU/10^6^ PBMC were considered positive.

To evaluate spike-specific proliferative responses, PBMCs (6×10^5^/200μl/well) were stimulated in triplicate with S and human actin peptide pools (15 mers, overlapping by 10 amino acids, Pepscan, Lelystad, The Netherlands) at a final concentration of 0.1 µg/ml for 7 days. After culture, cells were washed, stained with Live/Dead Fixable Violet Dye (Invitrogen, Waltham, Massachusetts, USA) and subsequently with CD3 PerCP 5.5 (BD Bioscience, Franklin Lakes, New Jersey, USA), CD4 APC-Cy7, CD8 FITC, CD25 PECy7 (all from BD Bioscience), and CD278 (ICOS) APC (Invitrogen). In some experiments, cells were permeabilized with cytofix/cytoperm (BD Biosciences), washed with perm wash (BD Biosciences) and stained with anti-perforin PE (Diaclone, Besançon, France, clone B-D48). Cells were fixed in PBS 1% paraformaldehyde and cell proliferation index (CPI) was determined by subtracting the percentage of CD25^+^ICOS^+^ CD3^+^CD4^+^ or CD3^+^CD8^+^ detected in PBMC incubated with actin peptides from the percentage of CD25^+^ICOS^+^ T-cell subsets detected in PBMC incubated with S peptides. A CPI ≥1.5% was considered positive (28). Flow cytometry analyses were performed with a BD FACS Lyrics flow cytometer and BD FACSuite software (BD Biosciences).

Memory B cells producing IgG antibodies (total and SARS-CoV-2 receptor binding domain (RBD)-specific) were evaluated using a commercial kit (MABTECH, ELISpot Path: Human IgG ALP). Results were given as number of RBD-specific or total IgG spots/10^6^ PBMC and values higher than 10 RBD-specific spots/10^6^ PBMC were considered positive. All the experiments were performed in the presence or in the absence of MMF (Sigma-Aldrich) in the cell culture medium at the concentration of 5 µg/ml.

### 2.7 Data collection and statistical analysis

Relevant clinical and experimental data for each patient (demographics, therapy, comorbidities, positivity to SARS-CoV-2 infection, IgG anti-spike protein quantification) obtained by reviewing clinical records or during an *ad-hoc* interview were stored in a dedicated encrypted database. Medians and interquartile ranges (IQR) were used to describe continuous variables, while percentages were used to express categorical variables. Continuous variables were compared with the Mann-Whitney U test, while categorical variables were compared with Pearson χ^2^ test. At univariate analysis we also calculated the odds ratio (OR) with their 95% confidence intervals (95% CI). Multivariate stepwise logistic regression models were built based on statistically significant variables identified at univariate analysis. Statistical significance threshold was set at 0.05 (two tailed). Statistical analyses were performed with Statistica for Windows release 12 (TIBCO Software Inc, Palo Alto, CA, USA) and MedCalc^®^ Statistical Software version 20.014 (MedCalc Software Ltd, Ostend, Belgium).

## 3 Results

### 3.1 Infection risk evaluation

In the first part of the study we obtained a complete 90 days follow-up for 119 out of 131 initially enrolled patients (21). Out of the 119 eligible patients for this follow up study, 114 took part to the survey at 6 months from the third dose vaccination. According to the data collected, 19 patients had SARS-CoV-2 infection confirmed by RT-PCR or antigenic test on nasal swabs, during the 6 months follow up period, accounting for a 16.7% infection risk. Interestingly, none of the patients developed a severe illness requiring hospitalization, as all the detected infections resulted in asymptomatic or mild disease (treated at home). Furthermore, we did not find any correlation between the observed infection risk and gender, as 16 out of 83 females (19.3%) and 3 out of 31 males (9.7%) developed SARS-CoV-2 infection (OR: 0.45, 95% CI: 0.12-1.66, p=0.2303).

To identify which variables were associated to an increased risk of infection following the third vaccine dose we performed a univariate statistical analysis (**Table 2**). Considering both clinical and pharmacological information available for the study cohort, we found that an ongoing immunosuppressive treatment with MMF was the only independent predictor of infection risk in our population.

**Table 2:** Univariate analysis of infection risk predictors following the third dose vaccination.

Predictors	No SARS-CoV-2 infection positivity	SARS-CoV-2 infection positivity	Odds Ratio	95% CI	p-value
AIH	16/79	4/15	1.32	0.39-4.49	0.6603
SSc	6/89	2/17	1.75	0.32-9.39	0.5165
Vasculitis	4/91	1/18	1.26	0.13-11.98	0.8383
RA	33/62	2/17	0.22	0.05-1.02	0.0524
Spondyloarthritis	22/73	2/17	0.39	0.08-1.82	0.2315
SLE	7/88	4/15	3.35	0.87-12.86	0.0779
CTD	16/79	6/13	2.28	0.75-6.89	0.1446
OLT	7/88	2/17	1.48	0.28-7.74	0.6430
CAD	5/90	1/18	1.00	0.11-9.08	1.0000
Hypertension	32/63	3/16	0.37	0.10-1.36	0.1343
DM II	9/86	4/15	2.55	0.70-9.34	0.1582
Neoplasia	5/90	1/18	1.00	0.11-9.08	1.0000
COPD	2/93	1/18	1.72	0.17-17.50	0.6459
PAH	2/93	1/18	2.58	0.22-30.03	0.4482
PDN	45/50	9/10	1.00	0.37-2.68	1.0000
MTX	26/69	3/16	0.50	0.13-1.85	0.2975
AZA	28/67	6/13	1.10	0.38-3.20	0.8547
HCQ	28/67	7/12	1.40	0.50-3.91	0.5262
**MMF**	**7/88**	**6/13**	**5.80**	**1.69-19.97**	**0.0053**
Leflunomide	7/88	0/19	0.30	0.02-5.52	0.4198
SSZ	5/90	1/18	1.00	0.11-9.08	1.0000
Abatacept	1/94	0/19	1.62	0.06-41.15	0.7716
Anti-TNF	8/87	1/18	0.60	0.07-5.13	0.6444
Anti-IL6	8/87	0/19	0.26	0.01-4.77	0.3670
Anti-IL17	4/91	0/19	0.52	0.03-10.09	0.6665
Calcineurin inhibitors	6/89	2/17	1.75	0.32-9.39	0.5165
Belimumab	2/93	0/19	0.98	0.04-20.77	0.9787

AIH, Auto-Immune Hepatitis; SSc, Systemic Sclerosis; RA, Rheumatoid Arthritis; SLE, Systemic Lupus Erythematosus; CTD, Connective Tissue Diseases; OLT, Orthotopic Liver Transplantation; CAD, Coronary Artery Disease; DM II, Type II Diabetes Mellitus; COPD, Chronic Obstructive Pulmonary Disease; PAH, Pulmonary Artery Hypertension; PDN, Prednisone; MTX, Methotrexate; AZA, Azathioprine; HCQ, Hydroxychloroquine; MMF, Mycophenolate Mofetil; SSZ, Sulfasalazine.

Bold text highlights the statistically significant results.

This result was confirmed also by the multivariate stepwise logistic regression, built considering variables with p<0.1 at univariate analysis, since MMF treatment was associated with an increased risk for SARS-CoV-2 infection (coefficient: 1.7582, standard error: 0.6307, MMF OR: 5.20, 95% CI: 1.70-20.00, p=0.0053; systemic lupus erythematosus, rheumatoid arthritis and age did not enter in the model).

### 3.2 Anti-SARS-CoV-2 antibody titer evaluation

Out of 119 eligible patients, 72 participated to the blood collection before and after the third dose vaccination for IgG anti-SARS-CoV-2 titer determination. The ELISA anti-spike antibody quantification before (t0) and after 30 and 90 days (t30 and t90, respectively) from vaccination showed that the new vaccine shot induced a rise in antibody titers, that reached and even overcame the antibody titers evaluated at the end of the primary vaccination cycle follow-up (**Table 3**).

**Table 3:** IgG anti-SARS-CoV-2 titers in the patients with a complete follow-up after the first booster administration.

Blood collection	Antibody titer (RU/ml)
Pre-vaccination (t0)	111.7 [44.0-172.3]
30 days after vaccination (t30)	182.3 [158.5-223.1]
90 days after vaccination (t90)	202.1 [149.2-256.9]

Median IgG anti-SARS-CoV-2 titers at different time-points (t0, t30 and t90) are expressed in RU/ml.

Antibody titer evaluation also highlighted that at the end of the 90-days follow-up after the third dose administration 71 out of 72 patients developed a detectable seroconversion (98.6%), while in only one of them persisted a lack of response to the vaccination (1.4%).

At 90 days from primary vaccination cycle, 19 patients out of 119 were classified as “non-responder” (21). Only 13 out of these 19 “non-responders” completed the 90-days follow-up in this second part of the study: interestingly, 12 of them developed a detectable immune response after the third vaccine dose. It is noteworthy that the only one patient with a persistent lack of seroconversion even after the third dose was an elderly woman affected by SLE, under treatment with MMF 1g/die and prednisone 5 mg/die, with different comorbidities, such as hypertension, type II diabetes, pulmonary arterial hypertension, and thyroiditis.

### 3.3 *In-vitro* evaluation of B- and T-cell response to MMF

In order to investigate the effect of MMF on B and T lymphocytes, *in vitro* experiments were carried out.

**Figure 2:**
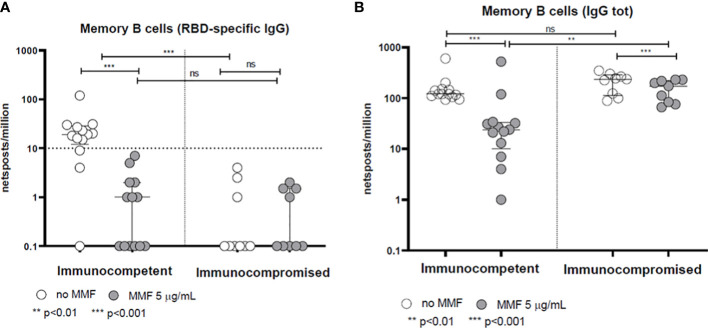
Spike-specific **(A)** and total **(B)** IgG memory B-cell response in immunocompetent and immunocompromised vaccinated subjects. Grey circles represent samples treated with MMF, while white circles represent untreated samples. The horizontal dotted line indicates the cutoff value for a positive response. **p<0.01, ***p<0.001, ns, not significant.

The frequency of memory B lymphocytes producing anti-Spike IgG (RBD) in healthy controls was reduced by *in vitro* treatment with MMF (p<0.001) (**Figure 2A**). Consistently, B lymphocytes isolated from patients during MMF treatment for their clinical condition show a low RBD-specific IgG production frequency (below the cut-off of 10 cells/10^6^ PBMC) at baseline even in the absence of *in vitro* treatment with MMF, and the difference between the production of specific anti-Spike IgG between treated and untreated B cells with MMF is not significant (p= not significant) (**Figure 2A**). Similarly, either in healthy controls and in patients, the frequency of total IgG-producing memory B lymphocytes was also decreased after *in vitro* treatment with MMF (p<0.001 for healthy controls, p< 0.01 for patients) (**Figure 2B**) while we did not observe a difference between the production of total IgG between B cells of patients and controls at baseline, i.e. without *in vitro* treatment with MMF (p= not significant) (**Figure 2B**).

**Figure 3:**
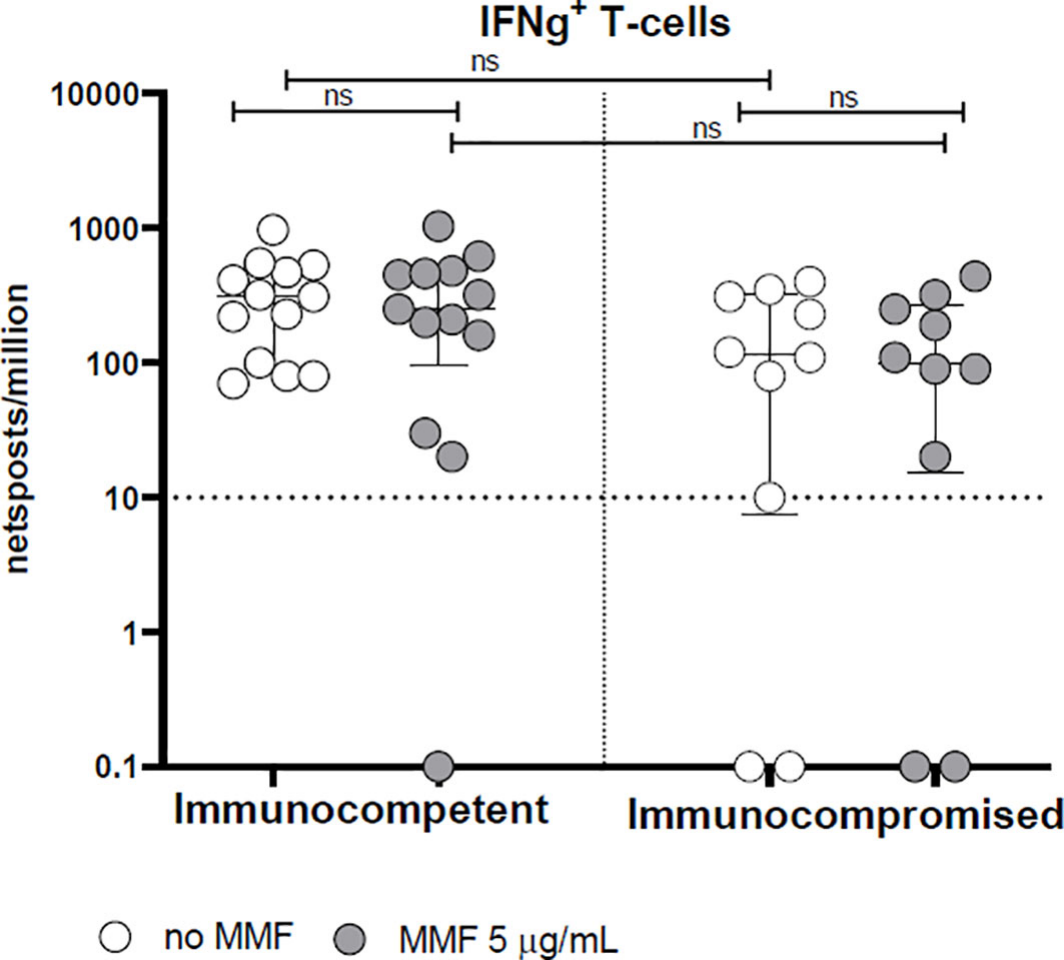
Spike-specific IFN-γ T cell response in immunocompetent and immunocompromised vaccinated subjects. Grey circles represent samples treated with MMF, while white circles represent untreated samples. The horizontal dotted line indicates the cutoff value for a positive response. ns, non-significant.

**Figure 4:**
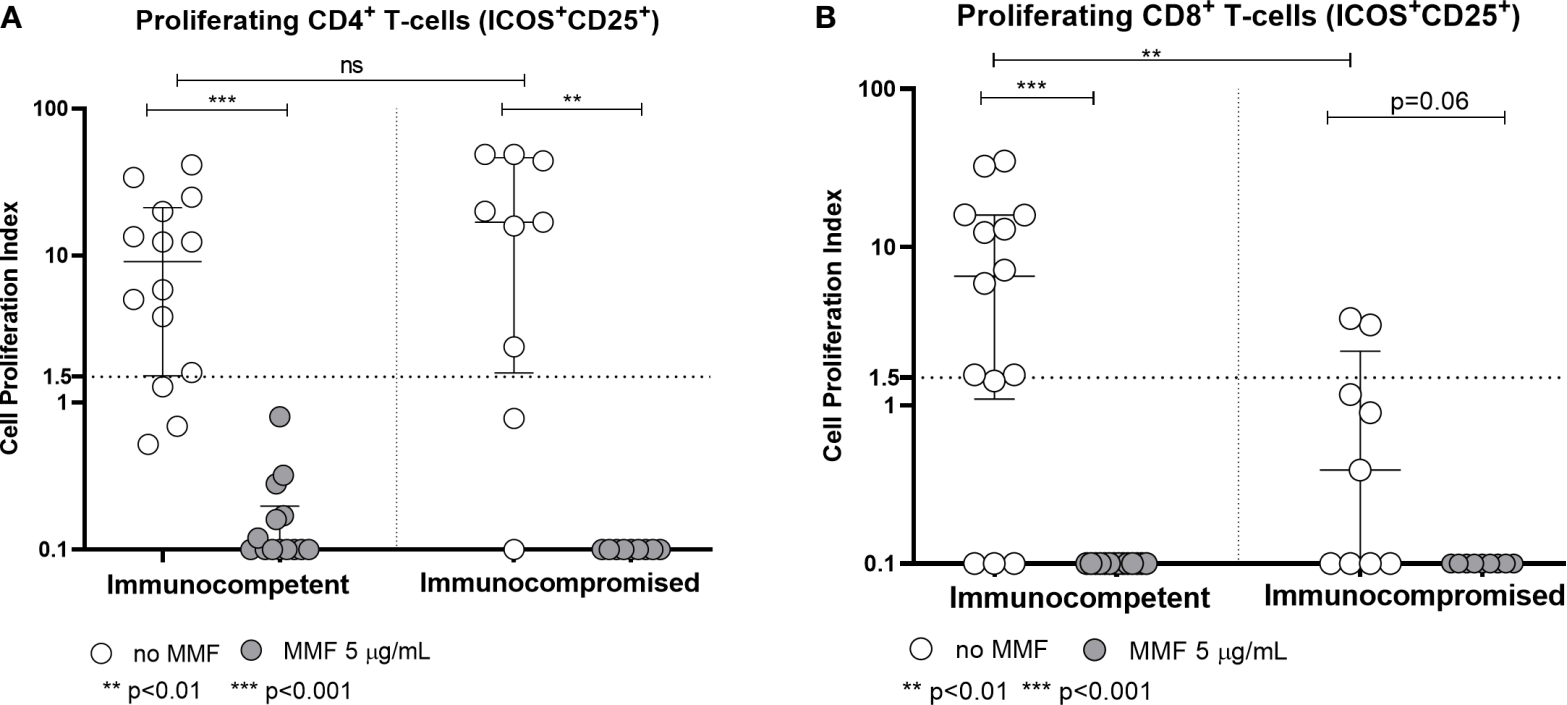
Spike-specific CD4^+^ **(A)** and CD8^+^ **(B)** T cell proliferative response in immunocompetent and immunocompromised vaccinated subjects. Grey circles represent MMF treated samples, while white circles represent untreated samples. The horizontal dotted line indicates the cutoff value for a positive response. **p<0.01, ***p<0.001, ns, non-significant.

With regard to the T-cell response, the rapid production of IFN-γ 24 hours after stimulation with Spike protein peptide-pool is not altered by MMF treatment either in healthy controls or in patients (p= not significant) (**Figure 3**). On the other hand, *in vitro* treatment with MMF reduced the lymphocyte proliferative index at 7 days; in fact, the proliferative response of CD4^+^ cells 7 days after stimulation with Spike protein peptide-pool is reduced in both controls (p< 0.001) and patients (p< 0.01) (**Figure 4A**). This is valid also for CD8^+^ lymphocytes in controls (p<0.001), while a not significant but consistent trend is evident also in patients (p=0.06) (**Figure 4B**). Furthermore, while the baseline CD4^+^ response (in the absence of *in vitro* treatment) is not significantly different between controls and patients (p= not significant), the CD8^+^ response is already reduced in patients receiving MMF for their clinical condition (p< 0.01) (**Figures 4A, B**).

As regards the comparison between the two groups of patients treated or not with MMF, no significant differences were found.

**Figure 5:**
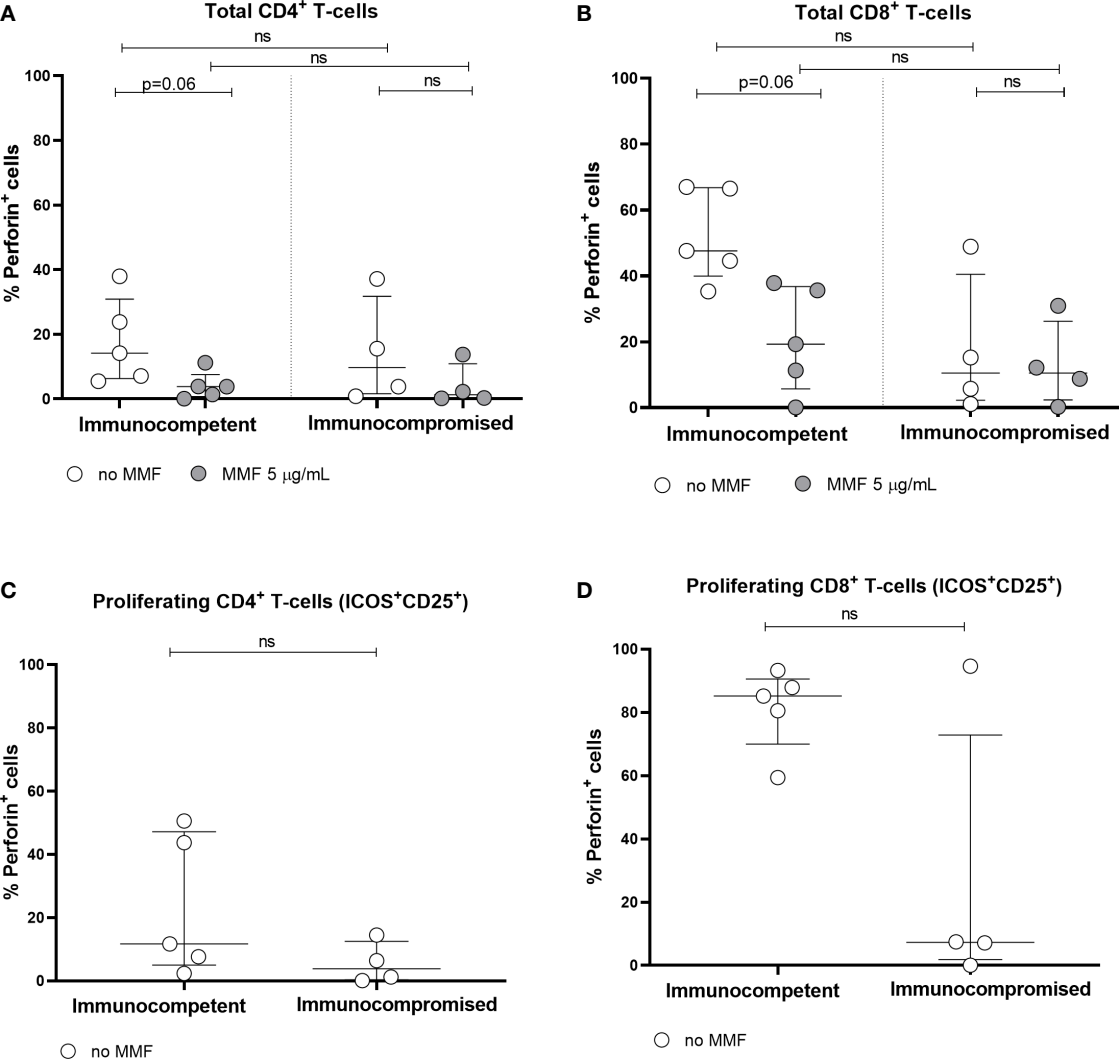
Perforin expression by total CD4+ **(A)** and CD8+ **(B)** T cells, and by spike-specific CD4^+^ **(C)** and CD8^+^ **(D)** proliferating T cells in immunocompetent and immunocompromised vaccinated subjects. Grey circles represent MMF treated samples, while white circles represent untreated samples. Perforin expression was not determined in MMF treated proliferating T cells, since proliferating T cells were not detectable after MMF treatment. The horizontal dotted line indicates the cutoff value for a positive response. ns, non-significant.

In 5 immunocompetent controls and 4 immunocompromised patients, perforin expression was determined in total and proliferating CD4^+^ and CD8^+^ T cells (**Figure 5**). Although the number of subjects tested was small, a trend towards a reduction in perforin expression in total CD4^+^ and CD8^+^ T cells was observed after *in vitro* treatment with MMF (**Figures 5A, B**). It was not possible to determine perforin expression in proliferating T cells after *in vitro* treatment with MMF, since proliferating T cells were almost undetectable. Moreover, immunocompromised patients receiving MMF *in vivo*, compared to immunocompetent controls, showed a trend towards a reduced perforin expression by both total and proliferating T cells even in the absence of *in vitro* treatment.

## 4 Discussion

Despite standard public health measures adopted all over the world during the ongoing pandemic, vaccines still represent the most powerful weapon to fight COVID-19. To date many studies have demonstrated, in real-world settings, high levels of effectiveness for the most diffuse vaccine platforms in protecting vaccinees from both infection and severe illness (13, 29–31). However, the observed waning in vaccine-induced immunity over time highlighted the need of booster doses to maintain an adequate protection level, especially in those individuals who are at greater risk of experiencing the most severe COVID-19 manifestations (32, 33). The observed waning in vaccines efficacy is mainly explained by their mechanism of action (33–35).

The available literature highlights that while 6 months after the conclusion of the primary vaccination cycle vaccines’ effectiveness in protecting from severe disease and hospital admission is still high, their ability to protect vaccinees from infection wanes with time, thus supporting the need of booster doses to guarantee a high protection level, especially after the emergence of new viral variants (36–38).

The need of booster doses is even more important in immunocompromised patients, who are known to be at higher risk to experience the most severe COVID-19 consequences, especially if we consider that a large proportion of subjects undergoing immunosuppressive therapy for autoimmune disorder management or to prevent organ rejection after solid organ transplant shows a reduced or even absent immune response to the complete primary vaccination cycle (21, 39–42).

In a previous work (21) we found that almost 24% of the immunocompromised patients initially enrolled in the Rivalsa cohort did not mount a detectable antibody response to the two-doses mRNA vaccination. Considering this relevant rate of vaccination failure and the known waning of vaccine-induced immune responses, we decided to evaluate the effect of the third dose in our cohort, in order to assess its ability to assure an appropriate protection to this at-risk populations.

Since the beginning of the pandemic, SARS-CoV-2 virus underwent evolution and adaptation processes resulting in the emergence of new viral strains. Different studies evaluated prevalent variants of concern (VOC) impact on vaccine effectiveness, highlighting that each VOC differentially affect vaccine-induced protection, mainly because of the polyclonal nature of the elicited responses (11, 43–45). Despite some recently emerged variants, such as delta and omicron, appear to be able to evade vaccine-related neutralizing antibodies, it is noteworthy that a complete mRNA-based vaccine schedule still assures a suitable protection especially against severe illness and death, while a booster dose administration should be necessary to increase neutralization activity and to gain protection against infection (36, 46–49).

According to ISS (Istituto Superiore di Santià – Italian health agency) data, in the timeframe of the study (October 2021 – April 2022) the prevalent SARS-CoV-2 variants in Italy were delta (since June to December 2021) and omicron (since January 2022 to the end of the study monitoring), two viral strains displaying a high transmission rate. Interestingly, despite the observed increase in antibody titers after the third vaccine dose, we observed a relevant percentage of infections, with no correlation to the previous seroconversion status. Moreover, the observed positivity did not result in severe illness, an observation in accordance with the report of Lawson-Tovey and colleagues, who highlighted that a major part of the infected patients fully recovered from COVID-19, supporting the protective role of vaccination in preventing severe or lethal outcomes in patients affected by inflammatory rheumatic and musculoskeletal diseases (32, 50).

Physiologically, host defenses against viral infections rely on mechanical barriers and innate immunity (mainly through Toll-like receptors signaling or DExD/H box RNA helicases) (51), and subsequently adaptive immunity responses activation (through the interaction of activated B cells and CD4^+^ T cells to initiate the process of somatic hypermutation and affinity maturation for the selection of long-lived high-affinity and antibody producing plasma cells and memory B cells) (52). In this scenario, antibodies intercept and bind the invading viral particles, while T lymphocytes recognize and destroy virus-infected cells. In particular, CD4 and CD8 T lymphocytes recognize viral antigens, fostering the activation and subsequent proliferation of effector and memory T cells (53), thus representing one of the most common targets of vaccine therapy. According to the known impairment of immune responses in patients undergoing immunosuppression, in this study we closely monitored their response to the third dose identifying as the only independent predictor of infection risk after vaccination, the treatment with MMF. Such observation is of great interest as MMF, the morpholinoethil ester prodrug of mycophenolic acid (MPA), is one of the most prescribed immunosuppressant drugs used to prevent graft rejection (especially in the case of kidney and liver transplantation) and to treat autoimmune diseases, such as lupus nephritis and other connective tissue diseases (54–57). The increased infection risk observed in our cohort could be explained by the pharmacological mechanism of action of this drug: MPA is a potent, reversible, selective and non-competitive inhibitor of the inosine monophosphate dehydrogenase, finally resulting in a reduction in B and T cells proliferation as well as in B lymphocyte produced antibodies (54–56, 58, 59). Moreover, MMF is also known to inhibit B cells and dendritic cells differentiation, thus further reducing immune responses (60–62). To better understand the reason why MMF-driven immunosuppression in spite of seroconversion is still linked to a higher risk of SARS-CoV-2 infection, we performed some ex-vivo experiments. Our data highlight that patients with ongoing MMF-treatment show a significant reduction in both total and specific anti-spike immunoglobulin production, as well as a reduction in CD4 and CD8 lymphocytes proliferation after *in vitro* stimulation with the spike protein. Moreover, we observed a trend towards perforin expression reduction, thus suggesting a reduced cytotoxic response, while IFN-γ production after 24 h stimulation with spike peptides was not altered. Considering that IFN-γ is produced by both CD4 and CD8 T cells, it could be hypothesized that this result could be explained by the already known ability of MPA to induce CD4 T cells reversible anergy and metabolic reprogramming (63). This proven effect of MMF treatment in reducing both humoral and adaptive cell mediated responses might explain the observed increase in in SARS-CoV-2 infection risk in MMF treated patients regardless the immunoconversion status, a data in agreement with the observed increase in re-hospitalizations for infective complications in transplant recipients treated with this drug (54, 56, 64, 65). Moreover, the observed lack of MMF effect on the rapid IFN-γ response could explain the observed asymptomatic or mild disease developed by the infected patients in our cohort.

Different studies have highlighted a suboptimal response to different vaccines, including the anti-SARS-CoV-2, in immunocompromised patients (21, 39, 40, 66–69). Interestingly, recently published papers have highlighted that, despite a weak response to the primary vaccination cycle, the booster dose administration to immunocompromised subjects is able to induce a strong immune response, resulting in antibody titers comparable to those of immunocompetent individuals (41, 70).

For 72 patients out 119 we were able to evaluate the seroconversion status, as they completed all the pre- and post-vaccination blood draws. By comparing their antibody titers at the end of the previous study follow-up (90 days after the first vaccine dose) with the ones detected after at least 6 months, before the third dose administration, we observed a waning in antibodies’ titers, as expected by the literature, where a decline over time in anti-SARS-CoV-2 IgG has been widely reported (12, 13). Taking into account the known weak response of immunocompromised subjects to vaccines, it is noteworthy that, in our cohort, the third vaccine dose administration resulted in a restoration of the immune response, even in those patients with an undetectable antibody titer at the end of the primary vaccination cycle. Such results are in accordance with the available literature about immunocompromised patients and booster anti-SARS-CoV-2 vaccination. One of the first papers dealing with this issue is the one of Shapiro Ben David and coworkers (71), describing a robust humoral response to the BNT162b2 booster vaccination in both immunocompetent and immunocompromised subjects, thus supporting the need of an additional vaccine administration after the complete primary cycle to assure a strong immune protection against COVID-19. Similar results were obtained also by Kontopoulou and colleagues in a smaller cohort of Greek adult immunocompromised patients, even if they observed a waning of the third dose-induced response after around 10 weeks from the booster, suggesting the need of an additional fourth dose (72). A positive immune response at the third vaccine dose, resulting in detectable antibody titers also in previously non-responders was observed also by Connolly’s and Aikawa’s research groups in two different cohorts of autoimmune patients as well as by Kamar and coworkers in a cohort of solid organ transplant recipients (17, 70, 73). These results, showing an increased humoral response after three vaccine doses were confirmed also by a recently published Norwegian prospective study (74), aimed to test a primary vaccination schedule based on three doses for immunocompromised patients, a study design that is slightly different from that of all the previously discussed ones, where the third dose was administered as a booster. Syversen’s research group followed prospectically a large cohort of patients with immune-mediated inflammatory diseases treated with immunosuppressive drugs and observed that such patients, that received the third vaccination 3 months after the second dose as per local protocol, developed at the end of this primary vaccination cycle an immune response that was comparable to that achieved in healthy controls undergoing the standard 2-doses primary vaccination cycle (74).

Finally, the persistence of a single non-responder after the first booster is in agreement with the available literature, where the persistent inadequate seroconversion after the additional vaccine shot correlates with stronger immunosuppressive schedules (69, 75).

This study is characterized by both strengths and limitations. In our opinion its most relevant strength is represented by the study prospectical design, with blood sample collection on a regular basis during the study duration, allowing a long follow-up evaluation, indispensable to evaluate both the humoral response trajectory evolution and the infection risk after the third dose in immunocompromised patients. On the other hand, we are aware of study limitations, mainly represented by the small sample size and by the telephonic survey adopted to collect data about the SARS-CoV-2 positivity after the third dose, which could have led to an underestimation of the real infection rate in our study population; anyway the possibility that this fact could affect the observed results is very limited. Furthermore, due to the local health policies, not establishing a routine identification of viral variants following SARS-CoV-2 infection positivity, it was not possible to draw any conclusion about the possible relationship between the timely prevalent variant and the observed infection risk, and consequently it was not possible to obtain any information about the ability of delta and omicron variants to evade immune protection induced by the currently available mRNA vaccines.

## 5 Conclusions

This prospective, observational study shows that a limited but relevant proportion of our immunocompromised subjects cohort develops SARS-CoV-2 infection after receiving the third vaccine dose and that the only independent predictor of infection risk is represented by ongoing MMF therapy. Moreover, we observed that the relevant proportion of inflammatory autoimmune diseases patients and liver transplantation recipients under immunosuppressive therapy not mounting a detectable humoral response 3 months after a complete primary vaccination cycle were able to respond to an additional vaccine administration, resulting, after 90-days follow-up, in an antibody titer comparable to that measured at the end of the primary vaccination cycle follow-up. Finally, it is worth of note that none of the infected patients experienced a serious disease, probably because their immune system resources (i.e. IFN-γ-mediated responses) were however sufficient to assure viral clearance, thus exceeding MMF inhibitory effect.

In conclusion, our results further support the observation that vaccines are of vital importance as a prevention agent in reducing the risk of infections in patients suffering of autoimmune diseases and/or undergoing immunosuppression. As a matter of fact, several studies highlighted that such frail population is at higher risk of developing bacterial (i.e., pneumococcus, haemophilus influenzae) and viral (i.e., seasonal influenza, herpes zoster, hepatitis) infections compared to the general population. Of note, the current clinical guidelines support the immunization of immunosuppressed patients with inactivated vaccines, while suggest a case-by-case evaluation for live vaccines use, as the risk-benefit balance is still in favor of vaccination also in this high-risk population. Furthermore, the correct timing for vaccine administration appears to be equally important, as it has been observed that to obtain an acceptable immune response along with minimal adverse effects, vaccinations should be scheduled during underlying autoimmune disease quiescence and, whenever possible, before immunosuppressive treatment (especially with high dose corticosteroids and biologics) initiation (76–79). As patients suffering from autoimmune diseases and organ transplantation recipients are usually treated with a variety of drugs inducing immunosuppression and considering that vaccine immunogenicity relies on both B- and T- cells mediated immune responses, when scheduling vaccinations for these patients it is also important to evaluate the potential effect of the ongoing therapy on vaccine response. Several studies showed an impaired vaccine immunogenicity in patients under immunosuppressive therapy, suggesting the need of a “drug washout” period or drug suspension prior to the vaccine administration. As the temporal window between the last immunosuppressive drug dose and vaccine administration is variable according to the ongoing pharmacological therapy, and could be as long as 6-12 months for rituximab, vaccination schedule should be tailored to the patient clinical context, according to the treating physician judgement, with the aim to achieve the better balance between individual patient risk of contracting vaccine preventable infections and the risk of under-treating the underlying autoimmune disease (80, 81).

In summary, a third vaccine dose in immunocompromised patients is effective in inducing a detectable humoral response also in those patients not responding to the primary vaccination cycle and is able to protect them from the most severe COVID-19 consequences, as demonstrated by the asymptomatic or mild symptomatic disease developed by infected individuals. These results support the need of repeated vaccination to support a sustained immune protection in patients treated with immunosuppressive drugs and in particular with MMF and the need to identify the appropriate timeframe for vaccine administration in order to optimize the vaccine-elicited immune protection.

## Data availability statement

The raw data supporting the conclusions of this article will be made available by the authors, without undue reservation.

## Ethics statement

The studies involving human participants were reviewed and approved by Comitato Etico Interaziendale Novara (CE 72/21). The patients/participants provided their written informed consent to participate in this study.

## Author contributions

Conceptualization: PS, DS, MR, ST, EZ. Formal *analysis:* PS, CB. Investigation: MR, ST, CB, EZ, EM, MC, ER, GC, DD, DC, RM, MB, LC, AC, MP, CR, DL, FZ, FB, DS, PS. Supervision: PS, DS. Writing original draft: MR. Review and editing: MR, ST, CB, EZ, EM, MC, ER, GC, DD, DC, RM, MB, LC, AC, MP, CR, DL, FZ, FB, DS, PS. All authors have read and agreed to the published version of the manuscript.
